# Stem Design in Total Hip Arthroplasty Influences Ipsilateral Knee Valgus: A Retrospective Comparative Analysis of 2953 Cases

**DOI:** 10.3390/jcm12206662

**Published:** 2023-10-21

**Authors:** Paul Thöne, Michael Stephan Gruber, Harald Kindermann, Walter Gussner, Patrick Sadoghi, Reinhold Ortmaier

**Affiliations:** 1 Medical Faculty, Johannes Kepler University Linz, 4020 Linz, Austria; 2 Department of Orthopedic Surgery, Ordensklinikum Linz Barmherzige Schwestern, Vinzenzgruppe Center of Orthopedic Excellence, Teaching Hospital of the Paracelsus Medical University, 5020 Salzburg, Austria; 3 Department of Marketing and Electronic Business, University of Applied Sciences Upper Austria, 4400 Steyr, Austria; harald.kindermann@fh-steyr.at; 4 Department of Orthopedics and Trauma, Medical University of Graz, Austria Auenbruggerplatz 5, 8036 Graz, Austria

**Keywords:** short stem, straight stem, total hip arthroplasty, valgus malalignment, knee arthritis

## Abstract

Background: Total hip arthroplasty (THA) affects the biomechanics of the hip and the patient gait. The stem design influences femoral lever ratios and tissue trauma. Biomechanical changes such as these have the potential to induce knee arthritis. A varus or valgus configuration of knee arthritis is formed by asymmetric loadings. The aim of this study was to evaluate the impact of stem design in THA on knee valgus by comparing a standard implant with an implant with a short stem. Methods: A total of 2953 patients who underwent primary total knee arthroplasty for end-stage osteoarthritis between 2015 and 2021 were included in this retrospective data analysis. Patients were divided into three groups, depending on hip status (straight stem, short stem, and native joint). Leg alignment was distinguished as varus or valgus, and the degree of axial deviation was measured. Descriptive and explorative statistical analyses were performed, with a *p* value < 0.05 set as significant. Results: Ipsilateral knee valgus occurred significantly more often in patients with straight stems (57.2%) than in those with short stems (29%) and native joints (25.8%) (*p* < 0.001). Additionally, mean valgus deviation was significantly increased in patients with straight stems (8.9°) compared to those with short stems (6.4°) or native hip joints (6.7°). Both findings were accentuated in women. Conclusions: Previous ipsilateral straight-stem THA is associated with knee valgus deformity, especially in women. Short-stem THA seems to be better suited to restoring physiological biomechanics and preventing the development of valgus osteoarthritis of the ipsilateral knee.

## 1. Introduction

Total hip arthroplasty (THA) is one of the most frequently performed surgeries in orthopedics, with a very high success rate. It has been awarded the title “operation of the century” [1].

THA can be performed via different approaches, and several stem and cup designs are available [2]. Cementless, standard straight stems are the most commonly used femoral implants [3]. For several years, the use of short femoral stems has increased and has been established as an alternative to straight stems with the potential for less bone loss and less invasive implantation [4].

In THA, restoring the natural biomechanics is key for function and implant longevity [5]. The stem design influences the ability to restore natural biomechanics, reduce soft tissue trauma, and position the femoral stem [6]. Deviation of lower-limb alignment, expressed as the hip–knee–ankle angle (HKAA) and the mechanical axis defined as the hip-center-to-ankle center line (HAL), are major influencing factors on knee pain and the development of osteoarthritis [7,8,9]. HKAA and HAL are influenced by stem design, muscular and soft tissue strength, and changes in the femoral lever ratio. Changes in these three influencing factors are proven risk factors for knee osteoarthritis [10].

Femoral offset influences biomechanics, pain, and function [11,12]. Loss of mediolateral offset alters HKAA and causes a lateral pass of the HAL at the tibia plateau [13]. This potentially increases the pressure in the lateral compartment of the knee [14,15]. Previous investigations have reported increased valgus malalignment in dysplastic hips after straight-stem THA [16,17,18]. Short-stem surgery is more soft-tissue sparing and bone saving [19]. Moreover, the restoration of native femoral offset (FO) and femoral antetorsion has been shown to be more accurate in short-stem THA [19,20].

The aim of this study was, therefore, to evaluate the impact of hip arthroplasty stem design on knee valgus by comparing a standard implant system to a short-stem system and to the native hip joint, with reference to the ipsilateral leg alignment. Further, potential alignment influencing reasons in THA are discussed. The research question was whether standard straight-stem systems had a greater impact on knee valgus than that of short-stem systems.

## 2. Material and Methods

### 2.1. Design and Subjects

A retrospective cohort analysis was designed. Subjects who underwent primary knee arthroplasty at the authors’ institution from 2015 to 2021 were investigated. The inclusion criteria were patients between the ages of 18–99 with end-stage knee osteoarthritis. A total of 2953 patients with a mean age of 72.5 years (SD = 9.2) were included—1883 (63.8%) women and 1070 (36.2%) men.

**Figure 1:**
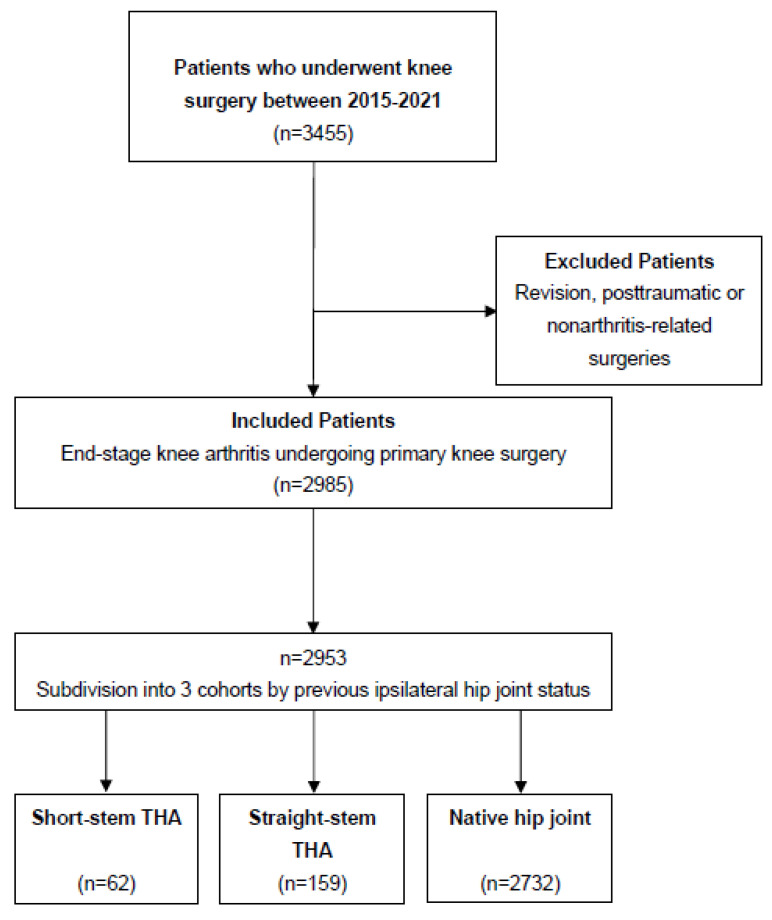
Inclusion process and investigation process in detail.

Other indications for knee arthroplasty, such as reoperations or posttraumatic arthritis, were excluded. Figure 1 illustrates the included and excluded cases and the reasons for inclusion or exclusion.

### 2.2. Data and Groups

All 2953 subjects were subdivided into 3 cohorts according to their hip joint status: 2732 (92.5%) patients had a native hip joint without osteoarthritis, 159 (5.4%) patients had undergone straight-stem THA, and 62 (2.1%) patients had undergone short-stem THA. Baseline characteristics, in detail, are provided in Table 1 to address potential confounding variables, which are considered in Section 3 and Section 4.

**Figure 2:**
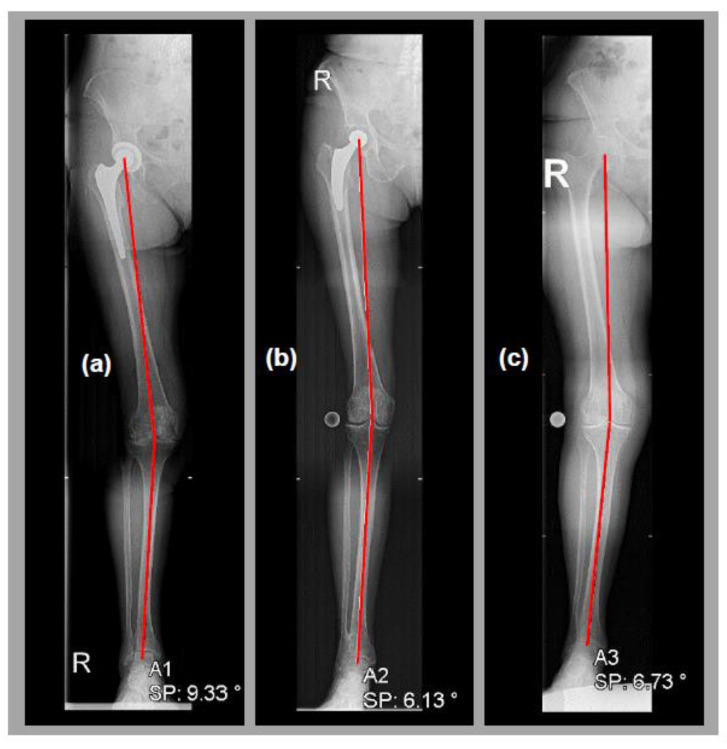
(**a**) Representative HKAA measurements in female subjects with straight stem, (**b**) short stem, (**c**) and native hip joint and ipsilateral knee valgus malalignment.

In these cohorts, we explored leg malalignment in terms of valgus or varus configurations shown on X-rays of the long leg axis. Furthermore, we quantified axial deviation as an absolute value of deviation, expressed as the hip–knee–ankle angle (HKAA) before knee arthroplasty (Figure 2). Additionally, gender and age were evaluated and correlated. The investigation did not consider arthritis-grading specifications, as the main criterion for consideration was the indication of endoprothetic knee restructuring.

In the uncemented standard straight-stem cohort, a Zweymüller stem (CBH stem; Mathys Ltd., Bettlach, Switzerland) was implanted via a lateral approach. In the uncemented short-stem cohort, an Optimys short stem (Optimys stem; Mathys Ltd., Bettlach) was implanted via posterior and anterior approaches. The index hip surgery took place in different orthopedic centers and was carried out by different surgeons. This was beneficial for randomizing a potential bias according to surgery skill and setting.

For radiological evaluation, the preoperative calibrated anterior–posterior X-ray in the long leg view in the standing position was used for measurements.

HKAA, defined as the angle between the mechanical axis of the femur and tibia, was calculated [21]. The Mikulicz line is a straight line from the femoral head rotation center to the middle of the talus. Valgus malalignment is defined as a lateral pass of the Mikulicz line at the HKAA knee point, while in varus malalignment, the Mikulicz line passes the medial knee.

### 2.3. Statistical Analysis

Explorative and descriptive statistical analyses were performed. Frequency distributions and summary statistics were calculated for demographic variables. For categorical variables, cross-tabulations were generated, and Pearson chi-square tests were used to compare distributions. For continuous variables, an analysis of variance (ANOVA) was used to examine differences in distribution between THA groups (no THA, straight-stem THA, short-stem THA).

Statistical analyses were 2-sided, and *p* ≤ 0.05 was considered statistically significant. The Bonferroni correction was used to address the problem of multiple comparisons. To evaluate the relationship between the type of THA and leg malalignment (valgus vs. varus), odds ratios were calculated. Pearson’s correlation coefficient was used to determine an undirected correlation between two metric variables. All statistical analyses were performed with IBM SPSS Statistics version 28.0 and R version 4.3.2.

### 2.4. Demographic Information

The majority of the patients were women (63.8%), and the mean age of the patients was 68.7 years at the time of knee surgery. The full demographic information is provided in Table 2.

## 3. Results

There were significantly more patients with valgus malalignment in the straight-stem group (57.2%) than in the short-stem (29%) and native-hip-joint groups (25.8%) (*p* < 0.001), as shown in Table 2. The short-stem group and the native-hip-joint group were not significantly different (*p* = 0.326).

A two-factor analysis of variance (independent variables: sex (female|male) and THA (native hip | short stem | straight stem); dependent variable: leg axis) showed significant differences in both main effects (sex: F = 4.13, *p* < 0.042; THA: F = 9.61, *p* < 0.001). The interaction effect between the two main effects was not significant (F = 1.72, *p* = 0.18).

Mean valgus deviation angles were significantly higher in the straight-stem group than in the short-stem group (*p* < 0.001) and the native-hip-joint group (*p* <0.001), as shown in Table 3. No significant difference was observed between the native-hip-joint and short-stem THA groups (*p* = 1.000).

The HKAA deviation in the valgus direction was significantly higher in straight-stem subjects and lower in short-stem subjects, compared to native-hip-joint subjects. This observation pertained to both men and women, while the absolute values of valgus deviation angle in all cohorts were less in men than in women. 

To evaluate the influence of THA on varus and valgus development for female and male subjects separately, a cross-table determination was calculated. Straight-stem THA in course of the valgus malalignment development was proved to be independent of gender (m: *p* = 0.007, f: *p* < 0.001). Short-stem THA and native hip joints had a high probability of not influencing knee malalignment (m: *p* = 0.409; f: *p* = 0.426).

**Figure 3:**
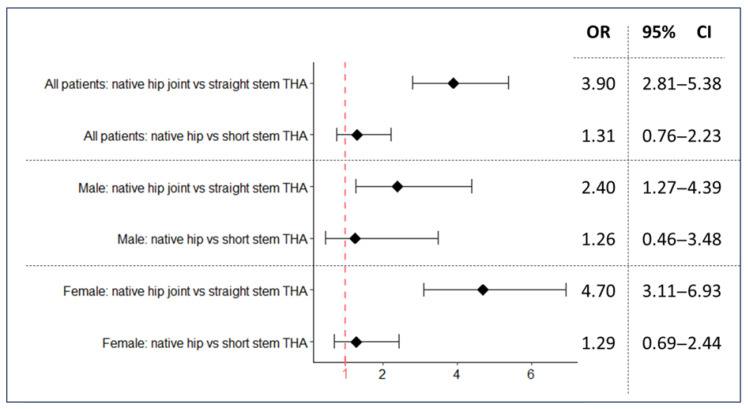
Hip stem design influences the odds ratio for valgus gonarthritis. OR > 1 indicates an increased occurrence of valgus, OR < 1 indicates a decreased occurrence of valgus.

The odds ratios for all combinations are shown in Figure 3. The results again showed that the use of a straight-stem design significantly increased the risk of developing valgus alignment, compared to short-stem THA and native hip joints.

The time period between THA and ipsilateral arthritic knee surgery was evaluated. In the straight-stem cohort, there was a mean duration of 142.4 months (SD = 83.8) in valgus subjects and 118.16 months (SD = 85.6) in varus subjects, with no significant difference (*p* = 0.076). In the short-stem cohort, there was a mean duration of 26.9 months (SD = 29.9) in valgus subjects and 39.7 months (SD = 34.3) in varus subjects, with no significant difference (*p* = 0.173).

Straight-stem THA subjects had a longer time period between THA and knee arthroplasty, as the implantation of short stems is a recent development in THA. There was a potential bias, as short stems were used more recently. To compensate for this discrepancy, those patients who had undergone THA and knee arthroplasty within comparable periods of time were considered. Two groups of short-stem and straight-stem patients with equal periods of time after index hip surgery, from 15 to 50 months, were compared. In these representative groups, a significantly greater valgus deviation was prevalent in the patients with straight-stem implants during this period (F = 4.608, *p* = 0.046).

### Effect of Stem Design on Knee Valgus

There is a significant effect of stem design on knee valgus. Straight-stem design leads to significantly more frequent valgus gonarthritis on the ipsilateral side and to increased mean valgus malalignment. 

## 4. Discussion

The aim of this study was to evaluate the impact of THA stem design on ipsilateral knee valgus. The hypothesis was that the impact of standard straight stems on knee valgus was greater than that of short stems. To the best of our knowledge, this is the first investigation examining the influence of previous ipsilateral THA on knee valgus malalignment formation.

**Figure 4:**
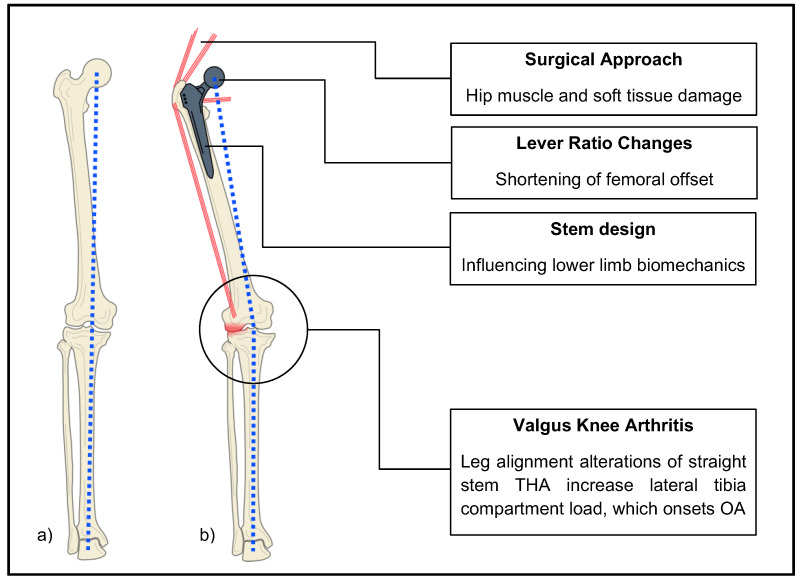
Impact of straight-stem THA on ipsilateral leg alignment and the association with valgus knee arthritis. (**a**) straight leg axis, (**b**) valgus malalignment, red lines symbolize muscle vector, HKAA is illustrated by blue dots.

We observed a significant increase in knee valgus malalignment in subjects with previous ipsilateral straight-stem THA, compared to native-hip-joint subjects. In addition, knee valgus malalignment rates in short-stem THA subjects hardly differed from those of native-hip-joint subjects. The deviation angle of the valgus malalignment was significantly higher in THA subjects than in native-hip-joint subjects, which highlighted our findings. The deviation angle in short-stem THA subjects was even more reduced than that in native-hip-joint subjects. Representative subjects are shown in Figure 2. Therefore, we deduced that previous ipsilateral straight-stem THA has the potential to influence knee valgus malalignment. The likelihood of knee valgus malalignment after straight-stem THA was three times higher than the likelihood of such malalignment after short-stem THA or for patients with native hip joints. As a result, we suspect that stem design, specific femoral lever ratio changes, and surgical approaches of straight-stem THA are pivotal influencing factors for knee valgus induction (Figure 4).

Concerning stem design, straight-stem THA configuration influences many biomechanical parameters in the ipsilateral leg, such as hip center medial shift and leg length discrepancy [22], hip internal rotation and patellar tilt [23], discrepancy in lower limb alignment [24], knee loading asymmetry [25], and gait changes [26], which persist more than two years after THA [27]. These alterations potentially promote valgus configuration of the knee joint.

Concerning changes in lever arm ratios in THA, femoral offset (FO) is essential. FO is closely related to HKAA [28]. Additionally, FO and muscular strength interact. There is a positive correlation between FO and hip abductor strength, soft tissue tightening, or muscular stability [29,30]. Therefore, a minor increase can be supportive, especially for postoperative weakness [31]. FO is reduced as a result of straight-stem THA in comparison to short-stem THA, which restores hip joint geometry more accurately [32]. As a consequence, a loss of FO increases lateral compartment load and causes asymmetric forces in the knee joint. Regarding hip rotator muscles, FO is suspected to weaken external rotators and strengthen internal rotators [33].

The surgical approach of THA influences many levels of the musculoskeletal system. Standard straight stems are conventionally implanted via a lateral transgluteal approach [34]. Soft tissue trauma affects the trophicity and strength of abductor muscles, such as the gluteus medius, the gluteus minimus, and the vastus lateralis. Surgery increases the fat ratio and decreases the volume of gluteal muscles, which leads to higher joint loadings [35]. The lateral approach causes the greatest damage to the abductor muscles and requires the longest postoperative recovery time [36,37]. Hip-muscle weakness is associated with knee OA [38]. Decreased hip abductor, extensor, and external rotator strength can lead to dynamic knee valgus [39,40]. Hence, the straight-stem surgical approach is associated with knee arthritis and valgus configuration.

However, straight-stem THA has various impacts on the ipsilateral leg. After primary THA, the odds for subsequent knee arthroplasty range from 2.1% to 6.8% [41,42]. All observed changes caused by stem design, femoral lever ratio changes, and surgical approaches subsequently cause specific HKAA deviations and loadings [22]. Simultaneously, these specific changes are risk factors for OA development [10]. In valgus knee OA, these changes occur with a load accentuation on the lateral compartment of the tibia plateau. This is consistent with the lateralization of HKAA in ipsilateral straight-stem hips, which enhances the lateral load on the tibia plateau [43]. The formation pathways are shown in Figure 4.

We observed a significant valgus difference in gender. Female subjects had twofold higher odds for ipsilateral knee valgus malalignment after undergoing straight-stem THA than males, but there was no difference in those undergoing short-stem THA. This corresponds with the higher valgus prevalence in women than in men. Moreover, we observed a higher valgus angle deviation in women than in men in all three cohorts, with an increase in the straight-stem THA cohort (f = 9.4°, m = 7.5°). Thus, the dynamic component of valgus development was considered. Clinical observations of patients with straight-stem THA and characteristic internal rotation gaits led us to conduct this investigation. Women are exposed to greater external moments and local extrema of loadings during the gait cycle [44]. Women are more prone to valgus loadings in motion [45]. In addition, the bone mineral density is frequently decreased in women, which promotes arthritic joint degeneration [46]. The augmented increased knee valgus in women with straight-stem THA emphasizes the dynamic aspect of our hypothesis, consistent with the literature. We suspect that straight-stem alternated leg alignment leads to higher stress on the lateral tibia compartment as a predetermined vulnerable point in leg alignment, especially during dynamic stress and when combined with decreased bone mineral density. 

The interoperative period between index THA and knee arthroplasty is critical for the development of malalignment. Ipsilateral knee valgus associated with a straight-stem implant is 3.9 times more likely than valgus in the interoperative period after a short-stem implant. The results are consistent with our hypothesis, as we expected an increase in valgus alignment changes caused by chronic asymmetric knee loading. Short-stem THA systems were first implanted in approximately 2000 [47].

We compared leg alignment changes in patients with previous ipsilateral straight-stem and short-stem THA. As a result, we observed significantly increased odds for arthritic knee valgus alterations and significantly increased angle deviation in valgus conformation, with an accentuation of the findings in women. We identified stem design, specific femoral lever ratio changes, and surgical approaches of straight-stem THA as pivotal influencing factors. The typical load on the knee joint occurs via recurrent dynamic loading. Valgus conformation increases over time after straight-stem implantation. Therefore, we suspect that straight-stem THA can influence ipsilateral knee valgus conformation and further cause pronounced lateral tibia compartment load, leading to knee osteoarthritis. Simultaneously, we showed that short-stem THA is not associated with similar alterations and sustains native leg alignment. For a holistic understanding of the THA influence on ipsilateral knee valgus, further related parameters, such as the body mass index, the grading of knee arthritis, the surgeons’ competence, medications, femoral offset, or bone density measurements, can be addressed. To investigate the impact of THA on ipsilateral knee valgus formation more accurately, further studies are necessary.

## 5. Limitations

Our study had several limitations to consider. First, there was a potential confounder caused by the different time period between the expression of knee arthritis in the short-stem and the straight-stem cohorts. Short-stem THA has not been established as long as straight-stem THA and, therefore, it has had a shorter observation period. To enable a comparison, sub-cohorts were formed from straight-stem and short-stem patients who had comparable times of follow-up after index hip surgery. Second, the analysis was designed retrospectively and, therefore, we could not ensure the highest level of evidence. Third, there was no measurement of HKAA before hip surgery in THA subjects, which would have enabled more accurate data. 

## 6. Conclusions

Patients with straight-stem THA have increased odds of ipsilateral knee osteoarthritis valgus malalignment, with higher expressions of the deviation angle, compared to patients with native hips or with short-stem THA. These findings are more pronounced in women.

## Figures and Tables

**Table 1:** Baseline characteristics of 2953 patients subdivided into 3 cohorts.

Cohort	Native Joint	Short Stem	Straight Stem
No.	2732	62	159
Sex (female)	1728	41	114
Sex (male)	1004	21	45
Age	70.2 (9.8)	74.0 (9.8)	73.3 (8.8)
Months since THA	-	33.3 (32.9)	130.5 (85.4)

Ages and months since THA are provided as means, with standard deviations (SDs) in parentheses.

**Table 2:** Baseline characteristics of 2953 patients, including patients after implantation of straight-stem total hip arthroplasty, patients after implantation of short-stem total hip arthroplasty, and patients with native hip joints.

Variable	Overall	Varus	Valgus
No.	2953 (100)	2139 (72.4)	814 (27.6)
Sex (female)	1883 (63.8)	1289 (68.5)	594 (31.5)
Sex (male)	1070 (36.2)	850 (79.4)	220 (20.6)
THA			
Native joint	2732 (92.5)	2027 (74.2)	705 (25.8)
Short stem	62 (2.1)	44 (71.0)	18 (29.0)
Straight stem	159 (5.4)	68 (42.8)	91 (57.2)
Age [mean (SD)]	68.7 (9.6)	68.6 (9.4)	68.9 (10.2)

Values shown are no. (%) unless otherwise noted. SD = standard deviation.

**Table 3:** Results of the two-factor ANOVA displaying the mean sex-specific HKA-deviation.

Cohort	Sex	Mean	SD	N
Native joint	Female	6.851	4.749	1728
	Male	6.459	4.145	1004
	*p* value	0.030	-	-
	Total	6.707	4.540	2732
Short stem	Female	6.621	4.899	41
	Male	5.904	3.391	21
	*p* value	0.551	-	-
	Total	6.378	4.430	62
Straight stem	Female	9.416	6.422	114
	Male	7.490	4.274	45
	*p* value	0.070	-	-
	Total	8.871	5.945	159

Data presented as absolute numbers (°). SD = standard deviation.

## Data Availability

Data directly supporting this investigation can be obtained by contacting the corresponding author.

## Abbreviations

ANOVA	Analysis of Variance
FO	Femoral offset
HAL	Hip-center-to-ankle center line
HKAA	Hip–kKnee–ankle angle
OA	Osteoarthritis
SD	Standard deviation
THA	Total hip arthroplasty
